# 

**Published:** 1906-11-03

**Authors:** 


### NOTICE TO CORRESPONDENTS.

All MSS., Letters, Books for Review, and other matters intended for the Editor should be addressed The Editor, "The Hospital," The Hospital Buildings, 28 & 29 Southampton Street, Strand, W.C.

The Editor cannot undertake to return rejected MS., even when accompanied by stamped directed envelope.

### Notice to Advertisers and Subscribers.

All Advertisements, Orders for Copies of The Hospital, and Business Communications should be addressed to **THE MANAGER (Not to the Editor),** The Hospital, 28 & 29 Southampton Street, Strand London, W.C.

The Cost of Subscription, post free, is as follows:—Per week, 3½d.; per quarter, 3s. 3d.; per half-year, 6s. 6d.; per annum, 13s. Foreign and Colonial:—Per annum, 19s. 6d., payable, in all cases, in advance.

### MUSCLES AND NERVES

**An Atlas of the Superficial Muscles and the Principal Motor Nerves of the Human Body for the use of Students of Anatomy and Nurses.**

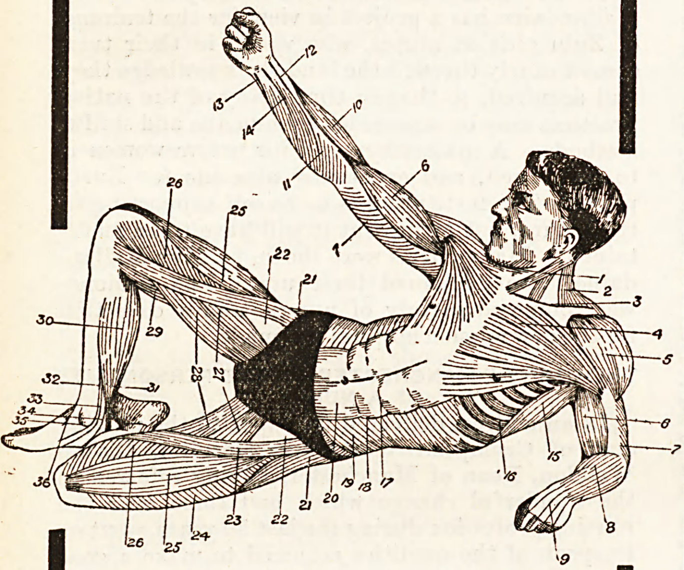

**By LOUIS B RAWLING, F.R.C.S.,**
*Assistant Surgeon and Senior Demonstrator of Anatomy, St. Bartholomew's Hospital.*

**Price 3/6 net, 3/9 post free.**

This Atlas contains a series of photographs of a well-developed male in such positions calculated to show off the superficial muscles to the best advantage. Opposite to each figure is a plan which not only illustrates the arrangement and direction of the muscles called into action, but also indicates the situation of the more important motor nerves.

The work cannot fail to be of the greatest practical utility to all Nurses and those who desire to gain a thorough insight into the superficial muscular formation of the human body, and obtain a general idea as to the position of the nerves which supply those muscles.

*Of all Booksellers, and of*
THE SCIENTIFIC PRESS, Limited,
28 & 29 SOUTHAMPTON STREET, STRAND, LONDON, W.C.

### POPULAR NURSING TEXT=BOOKS

THE BURDETT SERIES

**ONE SHILLING EACH**
**Post Free.**

The works contained in this Series are well written by expert teachers of the subjects of which they treat, and will form a very valuable and useful addition to a Nurse's Library. Each Volume is strongly bound in cloth cover, and contains about 100 pages.

**PRACTICAL HINTS ON DISTRICT NURSING.**

By Miss Amy Hughes.

**HOSPITAL AND PRIVATE NURSING.**
By Miss S. E. Orme.

**THE MIDWIVES' POCKET BOOK.**
**With Prefatory Chapter on the Midwives Bill.**
By Miss Honnor Morten, Author of "The Nurses' Dictionary," &c.

**FEVERS AND INFECTIOUS DISEASES:**
**Their Nursing and Practical Management.**
By William Harding, M.D. Ed., M.R.C.P. Lond., Author of "Mental Nursing," &c.

**NOTES ON PHARMACY AND DISPENSING FOR NURSES.**
New and Revised Edition. By C. J. S. Thompson.

**THE CARE OF CONSUMPTIVES.**
By W. H. Daw, M.R.C.S., L.R.C.P. Lond.

**MENTAL NURSING.**
By William Harding, M.B. Ed., M.R.C.P. Lond.

**SICK NURSING AT HOME.**
By Miss L. G. Moberly.

**GYNÆCOLOGICAL NURSING.**
By G. A. Hawkins-Ambler, F.R.C.S., Author of "The Commonwealth of the Body."

**SYLLABUS OF LECTURES TO NURSES.**
By Andrew Davidson, M.D.

THE SCIENTIFIC PRESS, LTD.

28 & 29 SOUTHAMPTON STREET, STRAND, LONDON, W.C.

